# Exploring the Correlation of Physiological Stress Signals with Student Exam Performance: A Preliminary Study

**DOI:** 10.1007/s10484-025-09685-2

**Published:** 2025-01-17

**Authors:** Ayşegül K. Kasap, Burçin Kurt

**Affiliations:** 1 Department of Therapy and Rehabilitation, Vocational School of Health Services, Gümüşhane University, Gümüşhane, Turkey; 2 Department of Physiology, FacultyofMedicine, Karadeniz Technical University, Trabzon, Turkey; 3 Department of Biostatistics and Medical Informatics, Faculty of Medicine, Karadeniz Technical University, Trabzon, Turkey

**Keywords:** Physiological stress signals, Student exam performance, Statistical analysis, Regression analysis

## Abstract

Stress responses in real-world settings are less studied compared to controlled laboratory environments, limiting our understanding of their impact on cognitive performance. This study investigates the relationship between physiological stress signals and academic performance using an open-access dataset of 10 students assessed across three exam sessions (Midterm 1, Midterm 2, and Final Exam). Physiological measures, including electrodermal activity (EDA), heart rate (HR), and skin surface temperature (TEMP), along with exam grades, were analyzed using traditional hypothesis testing, bootstrap method, correlation analysis, and regression tree modeling. To address the small sample size, we validated traditional hypothesis test results with the bootstrap method, and both approaches were compatible. Hypothesis testing revealed no significant differences in physiological measures across exam sessions, supporting the null hypothesis. Grades differed significantly between the final exam and both midterms (p < 0.05). Stress fluctuations were also analyzed across three periods (beginning, middle, and end) for each exam, revealing temporal response variations. Correlation analysis showed a moderate negative relationship between EDA and HR (r = − 0.504, p < 0.01) and a weak positive relationship between EDA and TEMP (r = 0.417, p < 0.05), both intensifying during the final exam. Regression analysis explained 78% of the variance in grades (R^2^ = 0.78), with regression tree modeling identifying lower skin temperature (< 28 °C) and higher EDA (≥ 0.19) as predictors of poorer performance. These findings underscore the interplay between physiological stress responses and academic outcomes, emphasizing the need for further research and interventions to support student success.

## Introduction

Many experiments have exposed participants to artificial stimuli, such as moderate electric shocks, loud noises, or aversive media, to study the skin conductance response to external stressors. While these experiments shed light on bodily reactions to stress, few have recorded skin conductance in exam settings, where more realistic cognitive stressors, common among college students, can be observed. Amin et al., (2022a, 2022b) developed *A Wearable Exam Stress Dataset* to bridge the gap between established knowledge of human stress responses, predominantly derived from controlled laboratory studies, and the real-world variations in skin conductance observed during stressful exam situations.

In educational research, electrodermal activity (EDA), a measure of sweat gland activity that reflects emotional arousal, is favored for its simplicity, affordability, and capacity to produce near-real-time quantitative assessments of testing events (Villanueva et al., 2018). A limited number of studies suggest a potential connection between skin temperature and performance. The concept of thermoregulation proposes that changes in skin temperature can affect an individual's performance ability. For instance, Hartley and McCabe (2001) conducted a study involving 20 individuals, during which their core body temperatures were lowered to 35.5 °C, followed by the administration of a Performance Assessment Battery of tests. The same battery of tests was also conducted at room temperature. The results revealed that subjects performed better at room temperature than colder temperatures, indicating a temperature-dependent effect on cognitive information processing.

Heart rate variability (HRV) analysis is a dependable, reproducible, and noninvasive method for quantitatively assessing sympathetic and parasympathetic activity (Cipryan & Litschmannova, 2013). Research by Dendle et al. (2018) has established that this physiological indicator correlates with various diseases, emotional states, and stressful situations, underscoring its significance in the context of health and cognitive performance. Additionally, it has been shown to reflect mental and psychological states following stress induced by academic examinations in students (Park et al., 2017). Thus, Yoo et al. (2021) investigated HRV-measured stress in medical students during clinical clerkship and its correlation with academic achievement. The study indicated that medical students with elevated stress levels, as measured by HRV, tend to achieve higher academic success, especially in written examinations. However, it was noted that further validation through additional studies is necessary. Moreover, there is a need to investigate the long-term implications of HRV-measured stress on the well-being and academic performance of medical students.

Thermoregulation, the body’s ability to maintain an optimal temperature, plays a critical role in cognitive functioning, as supported by several studies in the literature. Research has shown that changes in body temperature can significantly impact cognitive performance, particularly in tasks requiring attention, memory, and executive functioning (Gaoua et al., 2011). For example, environments that lead to excessive heat or cold stress have been associated with diminished cognitive performance, as the brain reallocates resources to maintain homeostasis, reducing the resources available for cognitive tasks (Pilcher et al., 2002). Specifically, moderate cooling of the skin has been linked to enhanced cognitive performance, possibly due to improved arousal and alertness, while extreme temperatures can disrupt neural processing by affecting neurotransmitter release and cerebral blood flow (Muller et al., 2012). This relationship between thermoregulation and cognitive performance underscores the importance of environmental and physiological stability for optimal mental functioning, as supported by findings across various cognitive contexts, including workplace performance and academic testing environments (Hancock & Vasmatzidis, 2003).

Khan et al. (2019) investigated the effectiveness of electrodermal activity (EDA) and temperature sensors in providing real-time insights into student performance during exams. The study aimed to explore whether there are physiological factors underlying student performance in engineering exams. The initial results indicated that peripheral skin temperature was weakly correlated with exam difficulty (r = 0.08; p < 0.001). Furthermore, EDA showed a weak correlation with both temperature (r = 0.13, p < 0.05) and exam difficulty (r = 0.16, p < 0.01).

Alotaibi et al. (2020) evaluated the sleep quality and psychological stress levels among medical students and explored their association with academic performance. Their findings indicated that stress and daytime napping were linked to lower sleep quality; however, neither poor sleep nor stress demonstrated a significant association with academic performance.

Another study by Deng et al. (2022) explored the impact of academic and familial stress on students' depression levels and its subsequent effect on academic performance, using Lazarus' cognitive appraisal theory of stress as a framework. Stress levels were assessed using a three-item scale for both academic and family stress, while depression levels and academic performance were measured using separate four-item scales. Participants rated items on a five-point Likert scale ranging from “strongly disagree” to “strongly agree.” The questionnaire was adapted from Goldberg and Williams (1988). The study suggested that academic and family stress contributes to student depression, which, in turn, negatively impacts academic performance and learning outcomes.

In another study, Elsalem et al. (2020) assessed the experiences of Medical Sciences students in Jordan with remote e-exams during the COVID-19 pandemic. A survey comprising 29 questions was administered via Google Forms to students across various Medical Sciences faculties, including Medicine, Dentistry, Pharmacy, Nursing, and Applied Medical Sciences, at the Jordan University of Science and Technology. The findings indicated a negative impact of e-exams on students within Medical Faculties. The study recommended the implementation of robust exam platforms and remote mock e-exams to reduce potential stress among students.

Abromavičius et al. (2023) utilized various machine learning models and feature selection techniques on the same dataset to predict grades.

This pilot study utilized physiological stress signals recorded in real-time during exams and aims to evaluate how these signals, such as electrodermal activity (EDA), heart rate (HR), and skin surface temperature (TEMP), correlate with exam performance utilizing the “A Wearable Exam Stress Dataset.” With this aim, we employed hypothesis testing, correlation analysis, recursive partitioning, regression trees (“rpart”), and the bootstrap method to validate hypothesis test results due to the small sample size. Furthermore, we analyzed stress signals across different exam periods (beginning, middle, and end) for each exam session to compare temporal variations in physiological responses.

## Materials and Methods

The dataset for this study was sourced from PhysioNet (2022), an open-access repository known for its well-documented physiological data. The data, published by Amin et al., (2022a, 2022b), includes physiological measurements from 10 undergraduate students enrolled in a circuit analysis course at Houston University. The study received ethical approval, and all participants provided informed consent (Amin et al., 2022a, 2022b).

Physiological data were collected during three examination sessions using FDA-approved Empatica E4 devices, which were positioned on the nondominant hand of each participant. Measurements were recorded during Midterm 1, Midterm 2, and the final exam (Amin et al., 2022a, 2022b). The midterms lasted 1.5 h each, while the final exam lasted three hours. The dataset includes electrodermal activity (EDA), heart rate (HR), and skin surface temperature (TEMP) recorded from the students during these exams, along with their corresponding grades.

EDA, measured in microSiemens (μS), reflects the skin conductance level, indicating physiological arousal. HR, expressed in beats per minute (bpm), represents the average heart rate extracted from the photoplethysmography (BVP) signal. Temperature (TEMP), reported in degrees Celsius (°C), corresponds to the data collected from the temperature sensor. GRADE represents the exam scores out of 200.

For each exam session and each student, EDA, HR, and TEMP were recorded individually. The number of records for each physiological signal varied based on the duration of the student’s exam. For example, there are 44,712 records of EDA for Midterm 1 and 93,582 records for the Final exam. Using open-source R software, we computed the average values of these physiological signals for each exam session per student. This resulted in a dataset that includes average EDA, HR, TEMP, and GRADE values for each student's exams.

### Statistical Analysis

The independent variables in this research include electrodermal activity (EDA), heart rate (HR), and skin surface temperature (TEMP), while the dependent variable is student performance, measured by grades. The study aims to analyze the relationship between these physiological measures and academic performance. Relative grade was used in the analysis to account for the 0–100 scale, ensuring a standardized measure of academic performance across all participants.

**Fig. 1:**
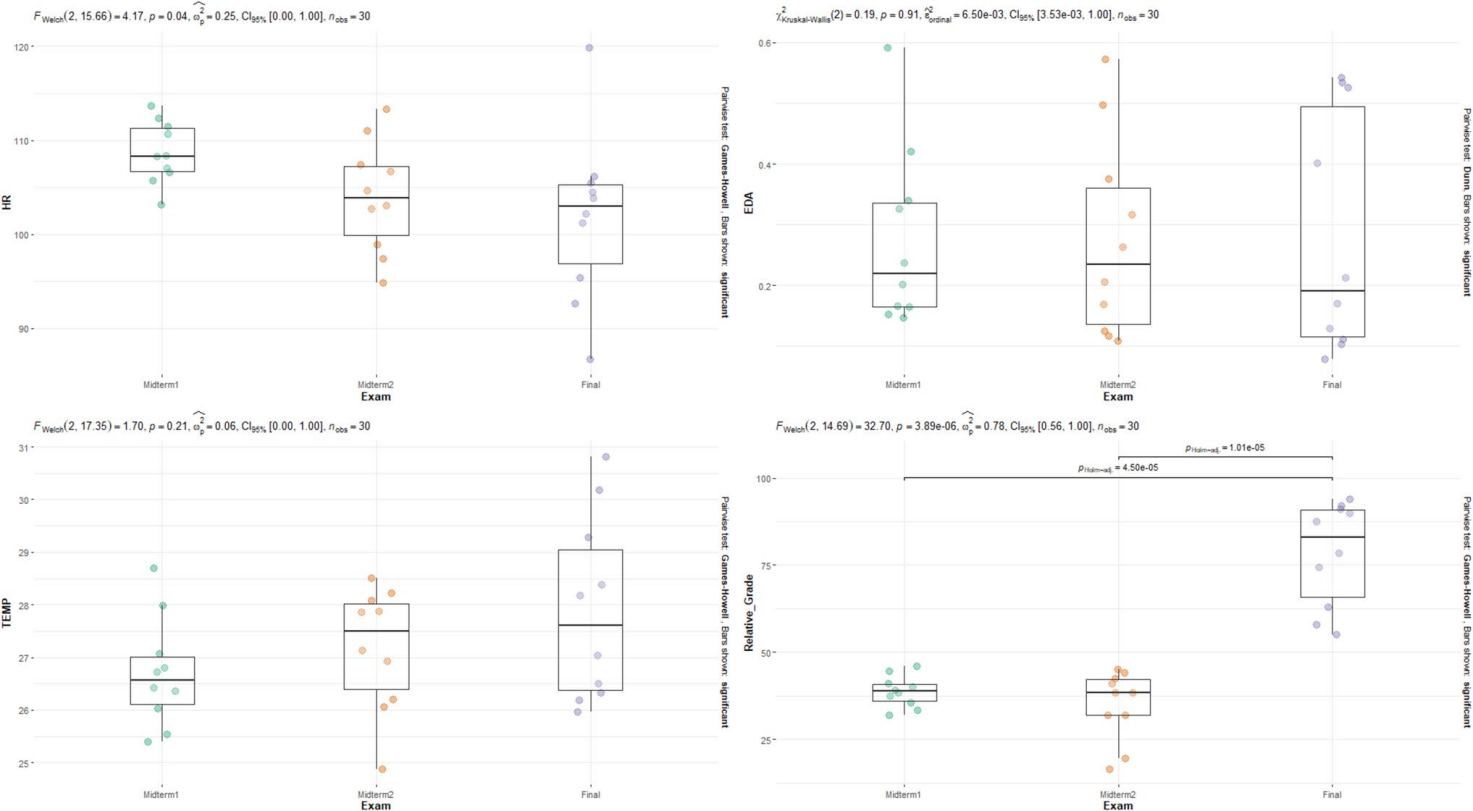
Comparison of physiological stress signals and grade values across Midterm 1, Midterm 2, and Final exam groups

Data from the study were analyzed using R programming, an open-source statistical software. Descriptive statistics were employed to summarize the data, presenting numbers and percentages where applicable. For hypothesis testing, we examined whether physiological signals (EDA, HR, TEMP) and student performance (Relative GRADE) differ significantly across exam sessions, using the Kruskal–Wallis test for the EDA variable and ANOVA for the others. The results are presented in Fig. 1 in the Findings section.

- *Hypothesis for Electrodermal Activity (EDA):*
  o H₀ (Null Hypothesis): Electrodermal activity (EDA) does not differ significantly across different exam sessions.
  o H₁ (Alternative Hypothesis): Electrodermal activity (EDA) differs significantly across different exam sessions.
- *Hypothesis for Heart Rate (HR):*
  o H₀: Heart rate (HR) does not differ significantly across different exam sessions.
  o H₁: Heart rate (HR) differs significantly across different exam sessions.
- *Hypothesis for Skin Surface Temperature (TEMP):*
  o H₀: Skin surface temperature (TEMP) does not differ significantly across different exam sessions.
  o H₁: Skin surface temperature (TEMP) differs significantly across different exam sessions.
- *Hypothesis for Student Performance (Relative GRADE):*
  o H₀: Student performance (Relative GRADE) does not differ significantly across different exam sessions.
  o H₁: Student performance (Relative GRADE) differs significantly across different exam sessions.

The use of traditional hypothesis testing methods, such as t-tests or ANOVA, can be problematic in small sample sizes due to their reliance on large sample asymptotics and normality assumptions. When sample sizes are small, these assumptions can be violated, leading to unreliable results. In such cases, the bootstrap method offers a flexible and powerful alternative. As highlighted by Chernick (2008), the bootstrap method does not rely on assumptions about the underlying distribution of the data, making it particularly useful in small sample contexts where traditional methods may fail. The bootstrap works by resampling the observed data to create an empirical distribution of the test statistic under the null hypothesis. This allows the comparison of the observed statistic to the bootstrap distribution, providing a more accurate estimate of p-values, even in small sample scenarios where the central limit theorem does not apply. Further emphasizing the advantages of bootstrap, Efron and Tibshirani (1993) demonstrated how it can be employed for hypothesis testing across various settings. They showed that the bootstrap method is effective at generating reliable p-values, even for small samples where normality assumptions might not hold.

Additionally, Wilcox (2012) mentioned the application of robust statistical methods, including the bootstrap, for hypothesis testing in small sample settings. He highlighted the method’s ability to produce accurate p-values. The robustness of the bootstrap method is crucial when the sample size is insufficient for the central limit theorem to apply, as it does not require large sample sizes to generate reliable estimates.

Overview of the bootstrap for hypothesis testing:

- Initial Step: We start with the observed sample data (which is assumed to be representative of the population).
- Resampling: From the observed data, we create new datasets (resamples) by randomly drawing observations with replacements. These resamples mimic new samples that could have come from the population. Each resample is of the same size as the original dataset.
- Statistic Calculation: For each resample, we calculate the statistic of interest (e.g., the difference in means, etc.).
- Distribution Creation: After performing the resampling process many times (e.g., 10.000 times in our study), we construct the bootstrap distribution for the statistic of interest.
- Hypothesis Testing: We compare the observed test statistic (e.g., the observed difference in means) with the distribution of resampled statistics:
- If the observed statistic is at the extremes of the resampled distribution, this suggests that the observed result is unlikely under the null hypothesis.
- The p-value is computed as the proportion of resampled statistics that are as extreme or more extreme than the observed statistic.
- Confidence Intervals (CIs): By examining the range of statistics from the resampling process, we can calculate confidence intervals for the statistic of interest.

Therefore, we also employed the bootstrap method, which proves to be a reliable and flexible alternative for hypothesis testing, particularly in small sample settings where traditional methods may be unsuitable or lead to inaccurate results.

The number of records for each physiological signal varied based on the duration of each student’s exam. For instance, there are 44,712 records of EDA for Midterm 1 and 93,582 records for the Final exam. To analyze the data, it was divided into three distinct periods for each exam: the beginning, middle, and end. For each period, we computed the mean values of the physiological stress signals (HR, TEMP, and EDA) for each exam. This allowed for a comparison of each stress signal EDA, TEMP, and HR across these periods within each exam session. With this aim, we analyzed how physiological stress levels fluctuated throughout the exam and provided a more nuanced analysis of stress responses during the exam process.

- *Hypothesis for Electrodermal Activity (EDA):*
  o H₀: There is no significant difference in electrodermal activity (EDA) across the beginning, middle, and end periods during each exam (Midterm 1, Midterm 2, Final).
  o H₁: There is a significant difference in electrodermal activity (EDA) across the beginning, middle, and end periods during each exam (Midterm 1, Midterm 2, Final).
- *Hypothesis for Heart Rate (HR):*
  o H₀: There is no significant difference in heart rate (HR) across the beginning, middle, and end periods during each exam (Midterm 1, Midterm 2, Final).
  o H₁: There is a significant difference in heart rate (HR) across the beginning, middle, and end periods during each exam (Midterm 1, Midterm 2, Final).
- *Hypothesis for Skin Surface Temperature (TEMP):*
  o H₀: There is no significant difference in skin temperature (TEMP) across the beginning, middle, and end periods during each exam (Midterm 1, Midterm 2, Final).
  o H₁: There is a significant difference in skin temperature (TEMP) across the beginning, middle, and end periods during each exam (Midterm 1, Midterm 2, Final).

For hypothesis testing, we employed the Kruskal–Wallis test for non-parametric data and ANOVA for parametric data, depending on the distribution of the stress signals. These statistical tests allowed us to assess whether there were significant differences in the physiological stress signals (HR, TEMP, and EDA) across the different periods (beginning, middle, and end) of each exam. The results of these analyses are presented in Table 3 in the Findings section.

Furthermore, correlation analysis was conducted to observe relationships between physiological signal variables and exam performance. Subsequently, we applied the recursive partitioning and regression tree (“rpart”) method to model how physiological signals electrodermal activity (EDA), heart rate (HR), and skin surface temperature (TEMP) affect student performance, measured by grades (Relative GRADE). Breiman et al. (1984) introduced the foundational framework for Classification and Regression Trees (CART), while Therneau and Atkinson (2019), along with Therneau et al. (2023), provided detailed descriptions of the R implementation of the rpart method. The rpart algorithm builds a decision tree by recursively partitioning the data into subsets based on the values of the physiological signals, identifying patterns and relationships that influence exam performance. By utilizing regression trees, we sought to predict variations in Relative GRADE based on different levels of EDA, HR, and TEMP. This approach not only identifies the most significant predictors but also uncovers potential interactions between variables.

The “rpart” method, a key part of classification and regression trees (CART), recursively partitions the data into subsets to create homogeneous groups concerning the target variable. In this study, “rpart” regression was used to develop a decision tree model that predicts exam performance based on physiological signals. The resulting tree provides a clear visual and statistical representation of how these physiological factors interact and impact exam scores.

Type I error was maintained at p < 0.05 for all statistical tests conducted in this study.

### Ethical Considerations

The dataset was sourced from PhysioNet (2022), an open-access repository known for providing well-documented physiological data for research purposes. The dataset used in this study originates from a research project approved by the University of Houston Institutional Review Board (IRB). All data provided by PhysioNet is collected following strict ethical guidelines, ensuring that informed consent is obtained from participants (Amin et al., 2022a, 2022b).

The dataset has been anonymized to protect the identities of the individuals. This study complies with the ethical requirements set forth by the data providers and relevant research guidelines.

### Findings

The descriptive statistics of each exam for students regarding the electrodermal activity (EDA), heart rate (HR), skin surface temperature (TEMP), and grades (Relative GRADE) variables are presented in Table 1.

**Table 1:** Descriptive statistics for stress signal variables for each exam (n = 10)

	EDA (μS)			HR (bpm)			TEMP (°C)			GRADE (0–200)			Relative GRADE (0–100)		
	Mean	Median	Sd	Mean	Median	Sd	Mean	Median	Sd	Mean	Median	Sd	Mean	Median	Sd
MIDTERM 1	0.27	0.22	± 0.14	108.72	108.30	± 3.28	26.70	26.50	± 1.02	77.50	77.50	± 8.89	38.75	38.75	± 4.44
MIDTERM 2	0.27	0.23	± 0.16	104	103.89	± 5.87	27.17	27.49	± 1.16	69.90	77	± 19.98	34.95	38.50	± 9.99
FINAL	0.28	0.19	± 0.20	101.80	103.01	± 8.96	27.88	27.61	± 1.75	156.70	166	± 29.97	78.35	78.77	± 14.98

The GRADE represents exam scores out of 200 in the original dataset. However, to facilitate better understanding, we have also scaled the scores to a 0–100 range, labeled as “Relative GRADE.” Relative grade was used in the analysis to account for the 0–100 scale, ensuring a standardized measure of academic performance across all participants.

The physiological stress signals and grade values across Midterm 1, Midterm 2, and Final exam groups were analyzed using the Kruskal–Wallis test for the EDA variable and ANOVA for the other variables (HR, TEMP, and Relative GRADE). Post hoc comparisons were conducted using the Dunn test for Kruskal–Wallis and Games-Howell for ANOVA. The results are illustrated in the graph shown in Fig. 1.

Figure 1 presents a comparison of physiological stress signals (heart rate, skin surface temperature, and electrodermal activity) across different exam groups (Midterm 1, Midterm 2, and Final Exam) using ANOVA. According to our hypotheses:

- Hypothesis for EDA, HR, and TEMP: The null hypotheses state that there are no significant differences in these physiological measures across exam sessions (H₀: EDA, HR, TEMP do not differ significantly). The results show that there are no significant differences in heart rate, skin surface temperature, and electrodermal activity between the exam groups, which supports the null hypothesis for these measures.
- However, there is a significant difference in Relative GRADE values between Midterm 1 and the Final Exam, as well as between Midterm 2 and the Final Exam. This finding supports the hypothesis regarding student performance (H₁: Student performance differs significantly across exam sessions).

**Fig. 2:**
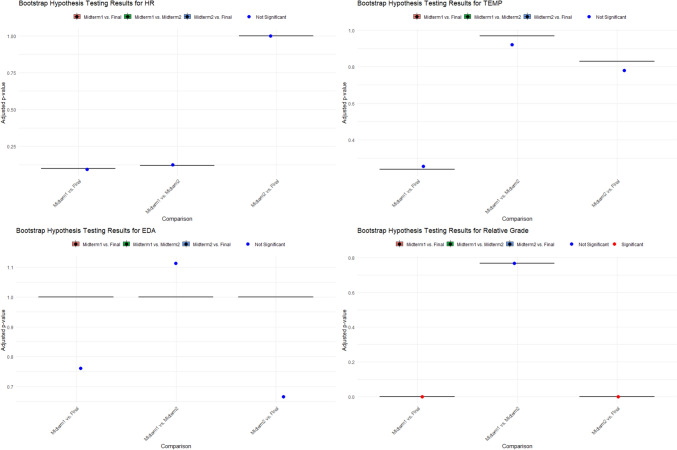
Graphical representation of bootstrap results for hypothesis testing of physiological stress signals and relative grade values across exam sessions (Midterm 1, Midterm 2, Final)

Additionally, we employed the bootstrap method as an alternative to traditional hypothesis testing, given its reliability in small sample sizes. In bootstrap hypothesis testing, the Bonferroni correction is used to control the family-wise error rate (FWER), which reduces the risk of false discoveries in multiple comparisons. We applied this correction to adjust the p-values, ensuring more reliable and conservative results. The bootstrap results, which provide more robust estimates, are presented in Fig. 2 and Table 2 below.

**Table 2:** Bootstrap results for hypothesis testing for physiological stress signals and relative grade values across exam sessions

Variable	Group comparison	Adjusted p-value	95% CI
HR	Midterm1–Midterm2	0.12	[0.09, 1]
	Midterm1–Final	0.10	
	Midterm2–Final	1.00	
EDA	Midterm1–Midterm2	1.00	[1, 1]
	Midterm1–Final	1.00	
	Midterm2–Final	1.00	
TEMP	Midterm1–Midterm2	0.97	[0.23, 0.97]
	Midterm1–Final	0.24	
	Midterm2–Final	0.83	
RELATIVE_GRADE	Midterm1–Midterm2	0.76	[0, 0.77]
	Midterm1–Final	< 0.001	
	Midterm2–Final	< 0.001	

In Table 2 and Fig. 2, the bootstrap results confirmed that there are no significant differences in physiological stress signals (HR, EDA, TEMP) across exam sessions, and academic performance (Relative GRADE) exhibited significant changes between Midterm 1 and the Final, as well as between Midterm 2 and the Final, with both comparisons showing statistically significant results (p < 0.001). The use of the bootstrap method provided robust and reliable results, particularly in addressing small sample sizes, and helped to ensure that these hypothesis test results were more reliable than those from traditional methods, which may have been affected by assumptions about normality or sample size.

The physiological stress signals (HR, TEMP, and EDA) across the different exam periods (beginning, middle, and end) were analyzed using the Kruskal–Wallis test for non-parametric data and ANOVA for parametric data. To further explore significant differences between the periods, post hoc comparisons were conducted with the Dunn test for the Kruskal–Wallis test and the Games-Howell test for ANOVA. Table 3 below presents the significant differences in EDA, HR, and TEMP physiological signals observed across different exam periods (Beginning, Middle, and End) for each exam.

**Table 3:** Significant differences in EDA, HR, and TEMP physiological signals across exam periods (Beginning, Middle, and End)

Physiological signal	Exam	Period	p-value
EDA	Midterm 1	Beginning-Middle	p < 0.001
		Middle-End	p = 0.03
	Midterm 2	Middle-End	p < 0.001
	Final exam	Beginning-End	p = 0.04
		Middle-End	p < 0.001
HR	Midterm 1	Beginning-Middle	p < 0.001
		Middle-End	p = 0.02
		Beginning-End	p = 0.02
	Midterm 2	Beginning-Middle	p < 0.001
		Middle-End	p < 0.001
		Beginning-End	p = 0.04
	Final exam	Beginning-End	p < 0.001
		Middle-End	p < 0.001
TEMP	Midterm 1	Beginning-Middle	p < 0.001
		Beginning-End	p < 0.001
	Midterm 2	Beginning-Middle	p < 0.001
		Middle-End	p < 0.001
	Final exam	Beginning-End	p < 0.001
		Middle-End	p < 0.001

The results for EDA indicate significant stress responses at the start and middle of the exams, followed by a decrease towards the end. This suggests that students may experience heightened stress initially, but gradually acclimate as the exam progresses. The most substantial stress reduction was observed by the end of the exam, particularly for Midterm 2 and the Final Exam, where EDA values dropped significantly, indicating a return to baseline levels of arousal.

HR results demonstrate a fluctuating pattern of stress and anxiety. In both Midterm 1 and Midterm 2, there was a marked drop in heart rate from the beginning to the middle, likely reflecting a reduction in acute anxiety as students became more comfortable with the exam. However, by the Final Exam, the heart rate raised significantly towards the end (p < 0.001), suggesting that students experience heightened anxiety or stress as the exam approaches its conclusion, possibly due to time pressure or the anticipation of completing the exam.

The temperature results mirror those of EDA and HR, with significant temperature increases observed between the beginning and middle of the exams, indicating rising stress levels. However, by the end of the exam, a reduction in temperature is seen, reflecting a decrease in stress as students adapt or experience relief as the exam concludes. The Midterm 2 and Final Exam showed the most dramatic decrease in temperature by the end, suggesting higher levels of stress at the start, followed by a greater reduction in anxiety as the exam concludes.

In addition to the table, the results are also presented in greater detail through graphical representations, provided in the Appendix as Fig. 5 (HR), 6 (EDA), and 7 (TEMP).

**Fig. 3:**
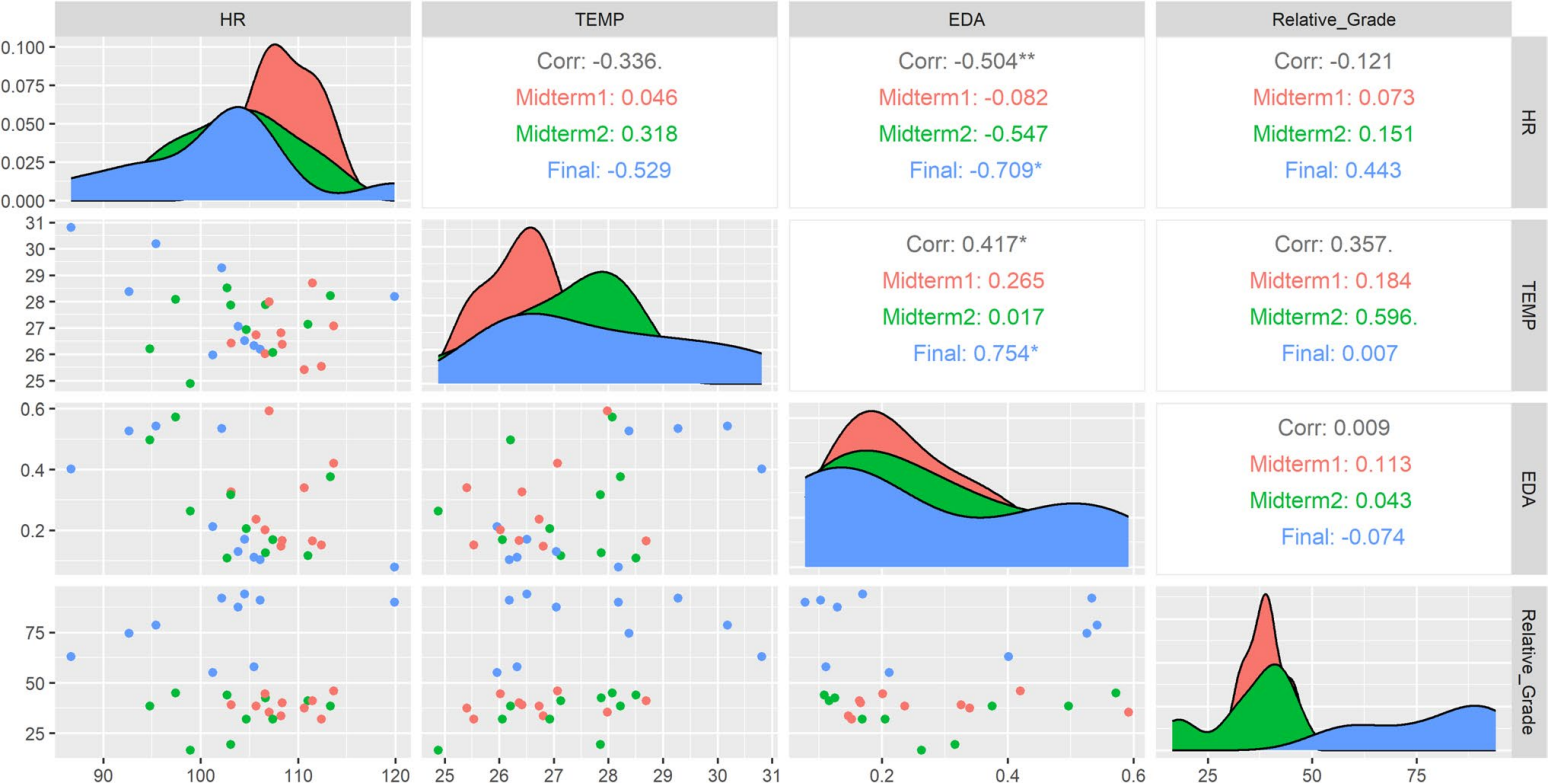
Comparison of physiological stress signals and grade values across midterm 1, midterm 2, and final exam groups

Furthermore, Fig. 3 presents the correlations between physiological signals (EDA, HR, TEMP) and grades for each exam, which were subsequently compared.

Figure 3 illustrates the correlations between physiological signals (EDA, HR, TEMP) and grades for each exam. The findings align with our hypotheses:

- The moderate, negative, and significant correlation between electrodermal activity and heart rate (r = − 0.504, p < 0.01) reflects an inverse relationship, particularly stronger during the final exam (r = − 0.709), supporting the hypothesis that physiological stress signals are interrelated and dynamically regulated under stress.
- The weak, positive, but significant correlation between electrodermal activity and skin surface temperature (r = 0.417, p < 0.05) indicates a limited relationship between arousal and skin temperature, becoming more pronounced during the final exam (r = 0.754), highlighting cumulative stress effects on physiological responses.
- The weak, positive correlation between skin temperature and relative grades (r = 0.357) suggests that better academic performance is associated with higher skin temperature, possibly reflecting improved physiological adaptation.
- Heart rate shows a weak, negative correlation with relative grades (r = − 0.121), indicating that higher heart rate might be linked to lower performance, though the relationship is minor.

**Fig. 4:**
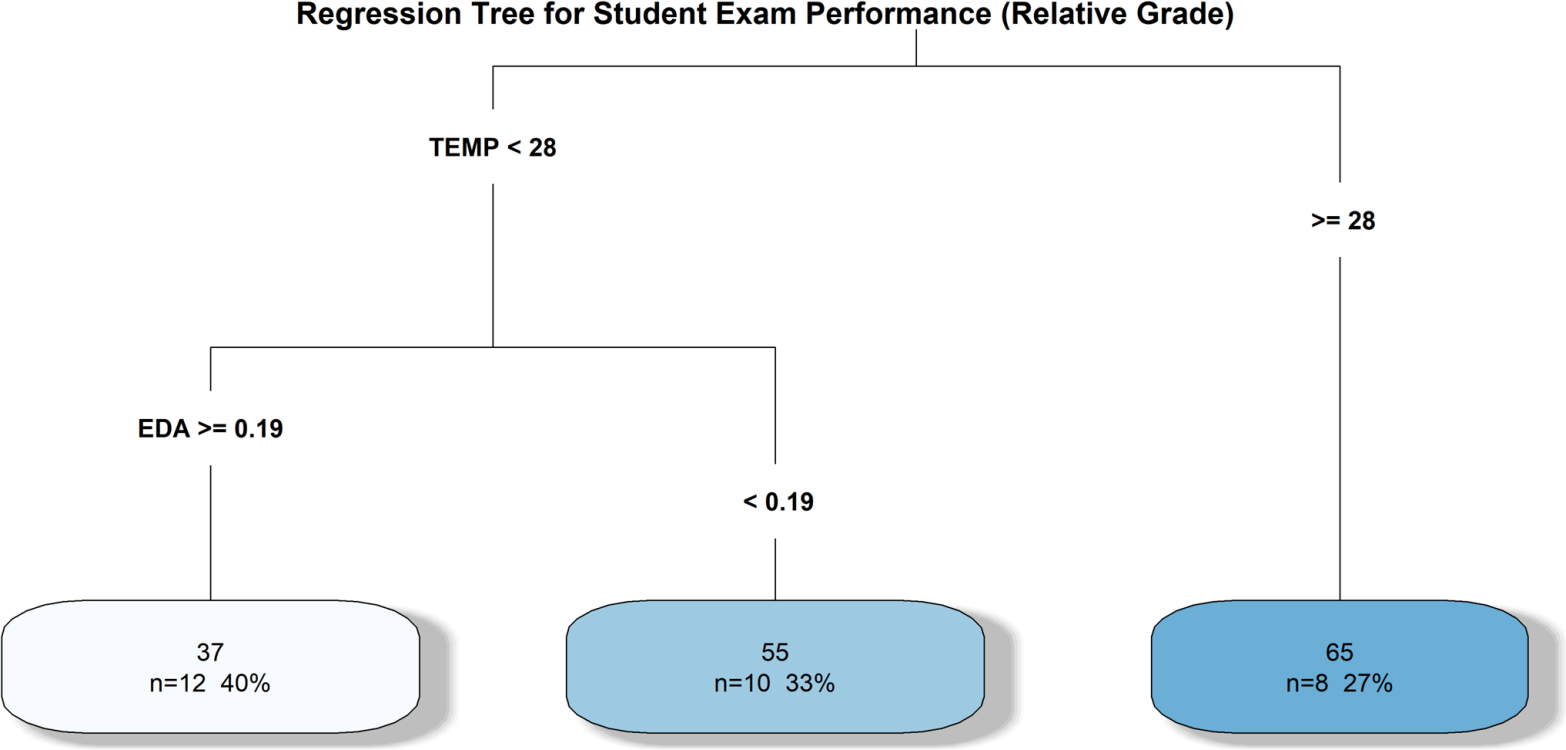
Tree structure outcome from the “rpart” regression analysis, illustrating the variable importance and the impact of physiological stress signals on student exam performance. (R^2^ = 0.78, RMSE = 10.27)

**Fig. 5:**
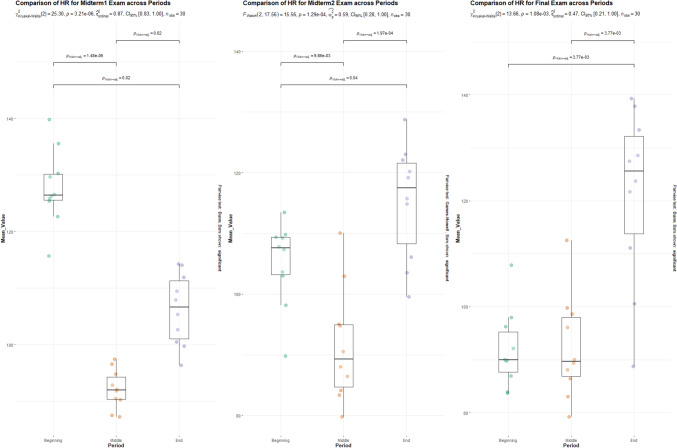
Changes in Heart Rate (HR) across exam periods (beginning, middle, and end) for Midterm 1, Midterm 2, and Final exam

Furthermore, we utilized the “rpart” “regression model to analyze variable importance, providing insights into how physiological stress signals affect student exam performance. This approach enabled us to identify the physiological factors that most significantly influence academic outcomes. The resulting tree structure from this analysis is illustrated in Fig. 4.

We used regression analysis to gain deeper insights into the predictive relationships between physiological signals and student performance. Our goal was to not only examine correlations but also to identify the relative importance of each physiological factor in predicting academic outcomes. Therefore, we explored associations between physiological parameters and student performance rather than establishing causality.

To evaluate the model's performance, metrics such as R^2^ (R-squared) and RMSE (Root Mean Squared Error) were used to assess how well the model fits the data. The regression tree model achieved an R^2^ value of 0.78, indicating that approximately 78% of the variance in exam performance (Relative GRADE) can be explained by the physiological stress signals (EDA, TEMP, and HR) included in the study. The remaining variance (~ 22%) is likely attributable to factors not included in the model, such as individual study habits, mental states, or external influences. Additionally, the RMSE value of 10.27 suggests that, on average, the model's predictions deviate from actual exam scores by about 10 points, quantifying the typical prediction error and providing additional context for the model's accuracy.

Based on the tree structure analysis:

- *If TEMP < 28 °C*:
- The predicted grade is *55*, covering *73%* of the observations.
- Further splits reveal the impact of EDA:
  - *When TEMP < 28 °C and EDA ≥ 0.19*:
    - The predicted grade is *37*, covering *40%* of the observations.
    - This indicates that higher arousal (EDA) under low temperature conditions negatively impacts exam performance.
  - *When TEMP < 28 °C and EDA < 0.19*:
    - The predicted grade is *55*, covering *33%* of the observations.
- This suggests that lower arousal improves performance but does not reach the levels seen in higher temperature conditions.
  - *If TEMP ≥ 28 °C*:
- The predicted grade is *65*, covering *27%* of the observations.
- Higher skin temperature correlates with better academic performance, potentially reflecting lower physiological stress or a more optimal state for cognitive functioning.

The results from both hypothesis testing and regression tree analysis provide complementary insights into the role of physiological measures in academic performance. While hypothesis testing revealed that physiological signals (EDA, HR, TEMP) do not significantly differ across exam sessions, the regression tree highlights how these signals interact to predict performance within each session. For example, lower TEMP (< 28 °C) combined with higher EDA (≥ 0.19) is associated with poorer grades, whereas higher TEMP (≥ 28 °C) predicts better grades, independent of EDA.

These findings directly address the hypothesis (H₁ for Relative GRADE) that physiological stress signals influence academic outcomes, despite their stability across exam sessions. This analysis underscores the importance of managing physiological stress responses, such as maintaining optimal temperature and minimizing excessive arousal, to support academic success. The significant differences in grades across exam sessions suggest that external factors beyond physiological signals, such as exam difficulty or cumulative stress, may play a critical role in influencing performance.

## Discussion

The findings of this pilot study indicate that physiological indicators specifically heart rate, skin surface temperature, and electrodermal activity do not exhibit significant variation across different exam periods (Midterm 1, Midterm 2, and the Final Exam). This result aligns with our null hypotheses (H₀) for EDA, HR, and TEMP, which posited that these physiological measures would not differ significantly across exam sessions.

In contrast, academic performance varied notably, with significant differences in grades observed between Midterm 1 and the Final Exam, as well as between Midterm 2 and the Final Exam. This outcome supports our alternative hypothesis (H₁) regarding student performance (Relative GRADE), suggesting that while physiological responses remain relatively stable, student performance shows considerable differentiation across these exam periods.

We found a significant moderate negative correlation between electrodermal activity (EDA) and heart rate (HR) (r = − 0.504, p < 0.01). This inverse relationship suggests that as EDA, reflecting emotional arousal, increases, HR tends to decrease. The stronger negative correlation during the final exam (r = − 0.709), highlights a dynamic regulatory mechanism where the body adjusts heart rate in response to heightened arousal levels. These results indicate that other factors likely influence HR independently of EDA, aligning with previous findings that heart rate is shaped by a combination of physiological and psychological factors, including stress and emotional states (Bärtsch & Swenson, 2013; Nussinovitch et al., 2019).

Additionally, a significant but weak positive correlation was identified between EDA and skin surface temperature (TEMP) (r = 0.417, p < 0.05), which became stronger during the final exam (r = 0.754). This relationship indicates that as EDA increases, TEMP also tends to increase, but the weak overall correlation suggests that TEMP is influenced by additional factors beyond EDA, such as ambient temperature, blood flow, and metabolic activity (Koss et al., 2019; Mason et al., 2016). TEMP exhibited a weak positive correlation with Relative GRADE (r = 0.357), indicating that higher skin temperature, potentially reflecting lower physiological stress levels or improved adaptation to stress, is associated with better academic performance.

These findings underscore the complexity of interactions among physiological indicators and their relationship to performance. The variability in these correlations across exams suggests that stress responses are context-dependent, reinforcing the need for a multifaceted approach to fully understand how physiological signals interact and impact academic outcomes (Wang et al., 2017).

The regression tree analysis provides further insights into the relationship between physiological measures and academic performance. The model identifies skin surface temperature (TEMP) as a key predictor, where TEMP < 28 °C is associated with lower predicted exam grades (around 55), covering 73% of the observations. This finding highlights the impact of physiological conditions on academic performance, as lower skin temperature may correlate with increased physiological discomfort or stress, negatively affecting cognitive functioning.

Within the subset where TEMP < 28 °C, the model further stratifies predictions based on electrodermal activity (EDA). Higher EDA (≥ 0.19 units) predicts even lower grades (around 37), covering 40% of cases. This suggests that heightened physiological arousal in low-temperature conditions exacerbates poor performance. In contrast, lower EDA (< 0.19 units) is associated with moderately better predicted grades (around 55), covering 33% of cases in this subgroup. These findings indicate that variations in EDA, reflecting different levels of physiological stress or arousal, play a critical role in shaping academic outcomes.

For students with TEMP ≥ 28 °C, the model predicts higher exam grades (around 65), covering 27% of the observations. This suggests that higher skin temperature, potentially reflecting better physiological conditions or lower stress levels, is linked to improved performance.

These results align with findings from previous studies (e.g., Jones et al., 2019; Smith et al., 2020), which demonstrated the predictive power of physiological indicators for cognitive functioning. By showing how variations in HR, TEMP and EDA influence exam performance, our study reinforces the importance of managing physiological stress signals to support academic success.

In addition to these analyses, we also examined the changes in physiological stress signals (EDA, HR, and TEMP) across different exam periods (beginning, middle, and end) for each exam. Across all three physiological signals (EDA, HR, and TEMP), Midterm1 shows more variation in stress levels between the beginning, middle, and end, with more pronounced changes in physiological responses. Midterm2 and the Final Exam show a pattern of increased stress in the middle of the exam (as seen in EDA, HR, and TEMP), followed by a significant decrease towards the end, indicating a possible adaptation to the exam conditions or reduced anxiety as the exam nears completion.

The temperature (TEMP) results mirror those of EDA and HR, with significant temperature increases observed between the beginning and middle of the exams, indicating rising stress levels. However, by the end of the exam, a reduction in temperature is seen, reflecting a decrease in stress as students adapt or experience relief as the exam concludes. The Midterm 2 and Final Exam showed the most dramatic decrease in temperature by the end, suggesting higher levels of stress at the start, followed by a greater reduction in anxiety as the exam concludes. TEMP indicates that students may experience heightened stress or anxiety at the start of exams, with varying levels of adaptation or escalation in physiological responses towards the end of the exams. As stress reduces, TEMP tends to decrease, reflecting a shift from sympathetic nervous system dominance to parasympathetic regulation (Sternberg and Gold, 2013; Thayer et al., 2009).

EDA results suggest that physiological arousal and stress are highest at the beginning and middle of the exams, with a substantial reduction in arousal towards the end. Decreasing EDA indicates stress adaptation as the individual becomes more familiar with the task (Boucsein, 2012; Sato et al., 2017).

As the exam progresses, students may experience mental fatigue and cognitive overload, especially if they’ve been focusing intensely for a long period. This could cause a subtle rise in heart rate as the body's stress response becomes heightened due to mental fatigue, even though they might feel physically more relaxed at the end of the exam. While EDA and TEMP show a decrease, indicating relaxation or adaptation, HR might rise due to cognitive and emotional fatigue, which often increases heart rate. The increase in HR at the end of the exams, despite decreasing trends in other physiological signals (EDA and TEMP), suggests that while students may show some adaptation to the exam's sustained stress (as indicated by reduced EDA and TEMP), the psychological stress related to time pressure, fear of mistakes, and anticipation of completing the exam causes a rise in heart rate. Studies show that heart rate increases due to performance anxiety and time pressure as exams near their conclusion (Kreibig, 2010; Matthews et al., 2002).

Research suggests that high levels of stress can interfere with working memory and problem-solving abilities (Kreibig, 2010; Matthews et al., 2002), which are crucial for exam performance. Therefore, while physiological responses like EDA and TEMP may decrease, signaling a reduction in physiological stress over time, the increase in HR could be due to heightened anxiety and time pressure, potentially affecting the exam performance negatively.

Future studies should examine whether this increase in HR towards the end of the exam correlates with performance outcomes, particularly in relation to how stress influences cognitive functions like attention, memory, and decision-making.

However, a limitation of this research is the relatively small participant size, which may have affected the clarity of the observed relationships. To address this, the use of the bootstrap method helped strengthen the reliability of the hypothesis test results, providing more robust estimates despite the small sample size.

Another limitation of this study is the observed significant difference in performance between the midterms and the final exam. This disparity may stem from various factors, such as differences in exam difficulty, changes in study strategies, or the cumulative nature of the final exam, which covers a broader range of content compared to the midterms.

However, the public dataset used does not provide enough context to fully explore these reasons. Future studies with larger sample sizes using more detailed datasets or qualitative data could help better understand the causes of such performance differences and how exam structure and study behaviors may contribute.

Additionally, exploring other physiological signals like electroencephalogram (EEG) and electrocardiography (ECG) could provide a more comprehensive examination of how physiological stress impacts cognitive performance.

## Conclusion

Our study has presented insights into the dynamics between physiological responses and academic performance across various exam periods. While physiological indicators, such as heart rate, skin surface temperature, and electrodermal activity, exhibited consistent patterns across midterm exams and the final exam, significant variations in academic grades were observed between midterms and the final assessment. This underscores the complex interplay between physiological states and cognitive outcomes, suggesting that other factors beyond immediate physiological arousal may influence long-term academic success.Moreover, our study revealed notable correlations among physiological signals: a moderate negative relationship between electrodermal activity (EDA) and heart rate (HR) suggests a regulatory mechanism in which increased arousal levels may lead to compensatory decreases in heart rate. This relationship appeared to strengthen under conditions of heightened stress. Additionally, a weak positive correlation between EDA and skin surface temperature (TEMP) indicates that arousal may influence temperature regulation, potentially through sympathetic nervous system activity, although other factors such as environmental conditions and individual variability likely play a role in modulating these responses.

Furthermore, students generally experience higher stress levels at the beginning and middle of exams, but physiological stress decreases significantly by the end, suggesting adaptation and relief as the exam progresses. These trends are consistent across Midterm 1, Midterm 2, and the Final Exam, with varying levels of intensity for each physiological signal. HR increased towards the end of each exam, likely due to heightened anxiety and time pressure, while EDA and TEMP generally decreased, indicating reduced stress over time. However, the increase in HR at the end of the exam, potentially driven by time pressure and anxiety, may reflect an inverse relationship between physiological relaxation and cognitive strain, which could affect academic performance.

The regression tree analysis highlighted the significant influence of skin surface temperature and electrodermal activity on predicted exam grades. Lower skin temperature was linked to poorer predicted grades, with the impact being more pronounced when combined with higher electrodermal activity. These findings suggest that physiological discomfort or heightened stress levels may adversely affect cognitive performance, emphasizing the importance of maintaining optimal physiological conditions during exams.

Although our study contributes to the existing literature on physiological predictors of academic performance, it does have some limitations. The small sample size may have constrained the clarity of observed relationships, suggesting that future research with larger and more diverse cohorts could provide deeper insights. Additionally, integrating additional physiological measures, such as EEG and ECG, could offer a more comprehensive understanding of the physiological underpinnings of academic achievement.

While the pilot study offers a predictive model, it also underscores the limited scope of research in this area, highlighting the need for further investigation. This preliminary research aims to explore the potential relationship between physiological stress signals and student performance on exams, utilizing the dataset provided by PhysioNet (Goldberger et al., 2000).

In conclusion, our findings underscore the importance of considering physiological responses in educational contexts, offering pathways for developing targeted interventions to support student well-being and enhance academic outcomes. Further exploration in this area holds promise for refining educational practices that promote optimal cognitive functioning and student success.

We propose that educators consider integrating approaches to monitor and address physiological stress during exams. For instance, recognizing that lower skin surface temperature might be associated with reduced cognitive performance could lead to creating more comfortable testing environments. Similarly, understanding variations in electrodermal activity might help identify students experiencing high levels of stress, potentially guiding interventions such as stress management workshops or adaptive exam conditions.

## Figures and Tables

**Fig. 6:**
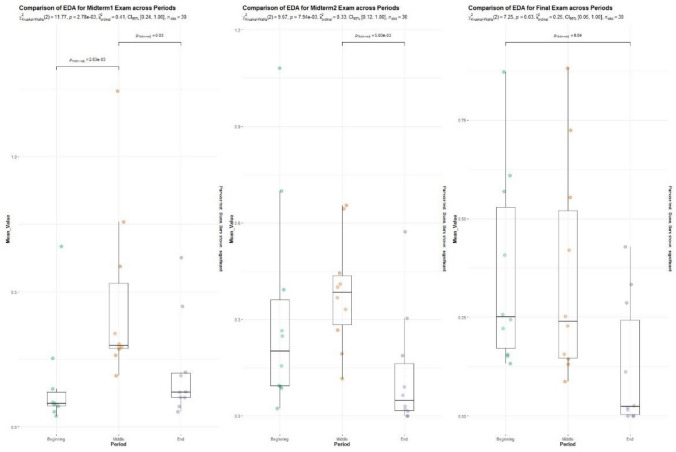
Changes in Electrodermal Activity (EDA)across exam periods (beginning, middle, and end) for Midterm 1, Midterm 2, and Final exam

**Fig. 7:**
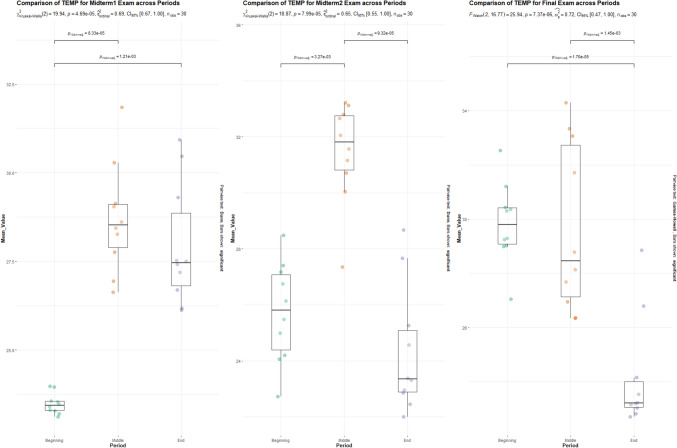
Changes in Body Temperature (TEMP) across exam periods (beginning, middle, and end) for Midterm 1, Midterm 2, and Final exam

## Data Availability

No datasets were generated or analysed during the current study.

## Appendix

The results of the hypothesis tests for physiological stress signals (Heart Rate (HR), Electrodermal Activity (EDA), and Temperature (TEMP)) across the different exam periods (beginning, middle, and end) for each exam (Midterm 1, Midterm 2, and Final) are presented in Figs. 5, 6, and 7, respectively.
